# Synthetic redesign of *Escherichia coli* for cadaverine production from galactose

**DOI:** 10.1186/s13068-017-0707-2

**Published:** 2017-01-21

**Authors:** Dong Hun Kwak, Hyun Gyu Lim, Jina Yang, Sang Woo Seo, Gyoo Yeol Jung

**Affiliations:** 1 School of Interdisciplinary Bioscience and Bioengineering, Pohang University of Science and Technology, 77 Cheongam-Ro, Nam-Gu, Pohang, Gyeongbuk 37673 South Korea; 2 Department of Chemical Engineering, Pohang University of Science and Technology, 77 Cheongam-Ro, Nam-Gu, Pohang, Gyeongbuk 37673 South Korea; 3 School of Chemical and Biological Engineering, Institute of Chemical Process, Seoul National University, 1 Gwanak-Ro, Gwanak-Gu, Seoul, 08826 South Korea

**Keywords:** Cadaverine, 1,5-diaminopentane, Galactose, Synthetic biology, Metabolic engineering

## Abstract

**Background:**

With increasing concerns over the environment, biological production of cadaverine has been suggested as an alternative route to replace polyamides generated from the petroleum-based process. For an ideal bioprocess, cadaverine should be produced with high yield and productivity from various sugars abundant in biomass. However, most microorganisms are not able to efficiently metabolize other biomass-derived sugars as fast as glucose. This results in reduced growth rate and low carbon flux toward the production of desired bio-chemicals. Thus, redesign of microorganisms is necessary for utilizing those carbon sources with enhanced carbon flux and product formation.

**Results:**

In this study, we engineered *Escherichia coli* to produce cadaverine with rapid assimilation of galactose, a promising future feedstock. To achieve this, genes related to the metabolic pathway were maximally expressed to amplify the flux toward cadaverine production via synthetic expression cassettes consisting of predictive and quantitative genetic parts (promoters, 5′-untranslated regions, and terminators). Furthermore, the feedback inhibition of metabolic enzymes and degradation/re-uptake pathways was inactivated to robustly produce cadaverine. Finally, the resultant strain, DHK4, produced 8.80 g/L cadaverine with high yield (0.170 g/g) and productivity (0.293 g/L/h) during fed-batch fermentation, which was similar to or better than the previous glucose fermentation.

**Conclusions:**

Taken together, synthetic redesign of a microorganism with predictive and quantitative genetic parts is a prerequisite for converting sugars from abundant biomass into desired platform chemicals. This is the first report to produce cadaverine from galactose. Moreover, the yield (0.170 g/g) was the highest among engineered *E. coli* systems.

**Electronic supplementary material:**

The online version of this article (doi:10.1186/s13068-017-0707-2) contains supplementary material, which is available to authorized users.

## Background

Cadaverine (1,5-diaminopentane) is an important platform chemical because it can be utilized to produce various important materials such as biopolymers, chelating agents, and other additives [1–3]. A promising polyamide, PA 5,10, whose properties are similar to nylon 6,6 can be polymerized from cadaverine with sebacic acid derived from plant oil [2]. The growing market size of biopolymers and bioplastics, which is expected to reach 5.08 billion US dollars by 2021 [4], also supports the importance of cadaverine production. Thus, development of an efficient process to produce cadaverine is strongly required, and various biomass-derived sugars should be utilized to fulfill the huge demand [5].

In nature, microorganisms produce cadaverine to adapt to changes in their environment, such as a drop of pH, for their survival [6]. Throughout the last decade, efforts have been made to enhance cadaverine production through metabolic engineering of industrial microorganisms. As an initial attempt, *Escherichia coli* has been studied for its tolerance of high concentrations of cadaverine [1]. This study demonstrated that *E. coli* is a suitable host to produce cadaverine because it can grow even in the presence of 20–50 g/L cadaverine. In addition, flux toward cadaverine synthesis was amplified by overexpression of enzymes including endogenous lysine decarboxylase (encoded by *cadA*) and disruption of degradation pathways. Furthermore, 9.6 g/L cadaverine was produced with a yield of 0.12 g cadaverine/g glucose and a productivity of 0.32 g/L/h [1]. Cadaverine production was further increased to 12.6 g/L with the expression of a synthetic small regulatory RNA mainly repressing *murE* [7]. A more recent study investigating systems metabolic engineering on lysine-producing *Corynebacterium glutamicum* demonstrated an industrial-scale application with a noteworthy production [2]. However, these promising results were based on utilization of glucose mainly obtainable from edible food sources, which may not be a sufficient feedstock for platform chemical production due to the possible impact on food supply and security [8]. Although there was an effort to engineer *E. coli* to directly utilize cellobiose obtainable from cellulose for cadaverine production [9], the production was relatively low (0.62 g/L) indicating that there is a significant room for improvement. Therefore, more studies on the utilization of various sugars from other inedible biomass are required to diversify feedstocks.

Production of cadaverine from galactose is quite promising because galactose can be easily found from the hydrolysate of macroalgae or dairy waste [10–12]. Particularly, macroalgae is not an edible biomass and does not require any fertilizer and arable land for its cultivation [13, 14]. Thus, it is expected that galactose can be a suitable feedstock to produce various platform chemicals in large quantities. However, the major drawback of industrial microorganisms such as *E. coli* is the slower utilization rate of galactose than that of glucose [15]. This low assimilation rate of galactose results in reduced rates of both growth and product formation even with well-performed glucose-dependent production pathways [16]. To overcome the limitation on galactose utilization, several combinatorial approaches have been demonstrated such as expression of several combinations of metabolic genes on *C. glutamicum* [17] and construction of fragmented chromosomal library perturbations for inverse metabolic engineering as well as a random mutagenesis approach on *Saccharomyces cerevisiae* [18, 19]. In a recent study, *E. coli* was re-designed by reconstruction of its utilization pathway with synthetic genetic parts including predictable promoters, 5′-untranslated regions (5′-UTRs), and terminators to achieve maximum expression [15]. The engineered strain showed a significantly enhanced growth rate (44.8%) and sugar utilization rate (53.1%), similar to glucose fermentation [15]. Furthermore, this engineered pathway was shown to be efficient when combined with the n-butanol production pathway [20].

In this study, we develop a novel *E. coli* capable of producing cadaverine from galactose, a promising future feedstock. To achieve this, we re-construct the entire galactose utilization and cadaverine production pathways with synthetic expression cassettes for maximum activity. Each gene is expressed under the control of synthetic promoters, rationally designed synthetic 5′-UTRs, and terminators at both transcription and translation levels. In addition, eliminating feedback inhibition of metabolic enzymes and competing metabolic pathways improve cadaverine production. In addition, our fed-batch fermentation demonstrates that *E. coli* is successfully re-designed for conversion of galactose into cadaverine.

## Results

### Design of synthetic expression cassettes for cadaverine production

**Fig. 1:**
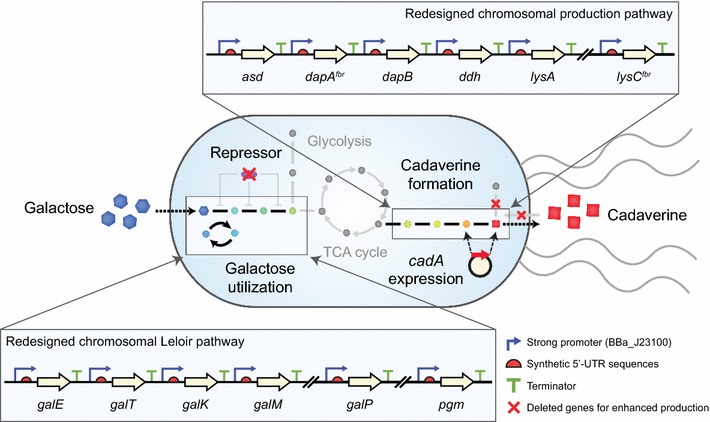
Overall strategy to develop the *E. coli* strain for cadaverine production from galactose. The native galactose metabolism was replaced through introduction of a re-designed Leloir pathway (*galE*, *galT*, *galK*, *galM*, *galP*, and *pgm*) on the chromosome. For cadaverine production, the carbon flux toward lysine was amplified by additional introduction of the re-designed production pathway (*asd*, *dapA* ^*fbr*^, *dapB*, *ddh*, *lysA*, and *lysC* ^*fbr*^) on the chromosome. Then, cadaverine was produced by expression of *cadA* (encoding lysine decarboxylase) on a high copy plasmid. The superscripts of two genes (*dapA* and *lysC*) indicate deregulation of feedback inhibition by site-directed mutagenesis

Biological synthesis of cadaverine is mainly achieved through decarboxylation of l-lysine, one of the essential amino acids [21]. In order to increase flux towards cadaverine production, we re-constructed the metabolic pathways of *E. coli* W3110 from aspartate to lysine by adding synthetic expression cassettes on chromosomes (Fig. 1). Additionally, instead of using 4 native enzymes (encoded by *dapD*, *argD*, *dapE*, and *dapF*), we used meso-diaminopimelate dehydrogenase (encoded by *ddh*) from *C. glutamicum* to directly convert 4-hydroxy-tetrahydrodipicolinate to meso-2,6-diaminopimelate. Moreover, the feedback inhibition of native enzymes was released by introducing point mutations based on previous studies (C352T on *dapA* [22] and C1055T on *lysC* [23]). Finally, a total of six genes (*asd*, *dapA* ^*fbr*^, *dapB*, *ddh*, *lysA*, and *lysC* ^*fbr*^) were chosen to be under the control of synthetic expression designs on chromosome (Fig. 1).

To express those genes, a strong constitutive promoter (BBa_J23100 from the Registry of Standard Biological Parts) was chosen because it does not require the addition of expensive inducers such as IPTG and its strong activity has been already adapted in the production of various value-added chemicals [24, 25]. In addition, synthetic 5′-UTRs for each gene were designed using UTR Designer to achieve maximum level at translation (Additional file 1: Table S1) [26]. Then, all genetic parts including promoters, 5′-UTRs, coding sequences, and terminators were assembled as a pseudo-operon in a plasmid (pACYC-Lys). Subsequently, this artificial operon was integrated into the chromosome as an additional copy by replacing *galR* to increase the galactose utilization rate [15]. For efficient recombination, *lysA*, which is next to *galR* in the genome, was also deleted. The cassette for *lysC* ^*fbr*^ was excluded in the artificial operon because we failed to obtain a proper positive colony during the cloning step. Possibly, harboring the constitutively expressing *lysC* ^*fbr*^ cassette along with other cassettes on the plasmid was a severe metabolic burden. Instead, we replaced the native chromosomal *lysC* cassette with the synthetic cassette of *lysC* ^*fbr*^ by homologous recombination.

Lastly, we expressed *cadA,* which converts lysine to cadaverine on high copy plasmids (pETduet) rather than chromosomal expression to increase the conversion efficiency similar to a previous study [1]. Originally, *cadA*-v1 (Additional file 1: Table S1) was designed as a 5′-UTR for *cadA* to achieve maximum expression. However, we did not successfully get a positive clone due to non-specific mutations around the promoter and 5′-UTR, as also observed in a previous study [1]. Therefore, we designed other versions of 5′-UTRs (*cadA*-v2 and *cadA*-v3, Additional file 1: Table S1) with lower expression levels. We obtained *cadA* with *cadA*-v3 whose predicted expression level was 30 times lower than that of *cadA*-v1.

### Enhanced galactose utilization for cadaverine production

**Fig. 2:**
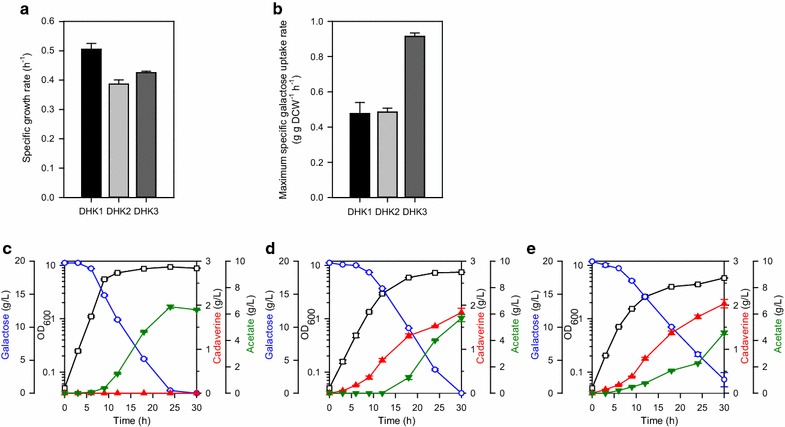
**a** Specific growth rate and **b** maximum specific galactose uptake rate of DHK 1–3 strains. One OD_600_ unit corresponds to 0.27 g dry cell weight (DCW)/L [15]. **c**–**e** Time-course fermentation profiles of DHK1-3 strains. The *left y-offset*, *right y-axis*, and *right y-offset* represents galactose, cadaverine, and acetate concentrations (g/L), respectively. The *left y-axis* represents OD_600_. The *x-axis* represents the culture time (h). The *error bars* indicate standard deviations of measurements from two independent cultures. Symbols: *open rectangle*, OD_600_; *open circle*, galactose; *closed upward triangle*, cadaverine; *closed downward triangle*, acetate

We first evaluated cadaverine production from galactose for the DHK1 strain harboring pET-cadA and the DHK2 strain with pET-cadA and synthetic expression cassettes for cadaverine production on chromosomes. The DHK1 strain grew relatively better than the DHK2 strain, indicating that expression of the synthetic cassettes for cadaverine production is a metabolic burden (Fig. 2a). The DHK1 strain did not produce any detectable amount of cadaverine despite the presence of all metabolic enzymes (Fig. 2c). However, the DHK2 strain produced significant amounts of cadaverine from galactose in terms of titer, yield, and productivity (Fig. 2d, 1.84 g/L, 0.0918 g/g, 0.0612 g/L/h, respectively).

We further engineered the native galactose operon (*galETKM*), *pgm*, and *galP* by replacing the original expression systems with synthetic expression cassettes. To achieve this, the synthetic galactose operon (*galE*-*galT*-*galK*-*galM*), *pgm*, and *galP* were introduced to their original loci on the chromosome based as previously published [15]. This DHK3 strain showed a slightly increased specific growth rate compared to the DHK2 strain (Fig. 2a) However, the maximum specific galactose uptake rate of the DHK3 strain (0.914 g/g DCW/h) was remarkably higher than that of both the DHK1 (0.477 g/g DCW/h, 192%) and DHK2 (0.485 g/g DCW/h, 188%) strains (Fig. 2b). Moreover, this strain produced cadaverine with higher titer (2.03 g/L), yield (0.102 g/g), and productivity (0.0678 g/L/h) than those of the DHK2 strain (Fig. 2e). This improvement indicates that galactose was successfully utilized and converted to cadaverine through our re-designed metabolic pathway with synthetic expression cassettes.

### Deletion of genes for cadaverine degradation and re-uptake

**Fig. 3:**
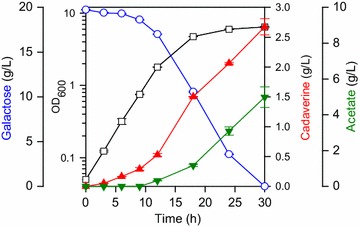
Time-course fermentation profiles of the DHK4 strain. The *left y-offset*, *right y-axis* and *right y-offset* represent galactose, cadaverine, and acetate concentrations (g/L), respectively. The *left y-axis* represents OD_600_. The *x-axis* represents the culture time (h). The *error bars* indicate the standard deviations of measurements from two independent cultures. Symbols: *open rectangle*, OD_600_; *open circle*, galactose; *closed upward triangle*, cadaverine; *closed downward triangle*, acetate

Our next step was inactivation of competing pathways to increase cadaverine production by preventing possible degradation and re-uptake of cadaverine. It is known that several enzymes whose substrates are diamines can degrade cadaverine because of structural similarities to cadaverine [1]. These enzymes are putrescine/cadaverine aminopropyl transferase (encoded by *speE*), spermidine acetyltransferase (encoded by *speG*), γ-glutamylputrescine synthetase (encoded by *puuA*), and cadaverine aminotransferase (encoded by *ygjG*) [1, 27, 28]. In addition, putrescine importer (encoded by *puuP*) might non-specifically import extracellular cadaverine into the cytosol [1]. As deletion of those five genes led to an increase in cadaverine production [1], we also removed them from the chromosome of the DHK3 strain, and this strain was designated as the DHK4 strain. We observed increased cadaverine titer and productivity from galactose to 2.67 g/L and 0.0892 g/L/h (Fig. 3), which was 31.5% higher than the parental DHK3 strain. The yield was also enhanced to 0.134 g/g, indicating effective cadaverine production with minimization of the degradation and re-uptake of cadaverine.

### Fed-batch cultivation of the DHK4 strain

**Fig. 4:**
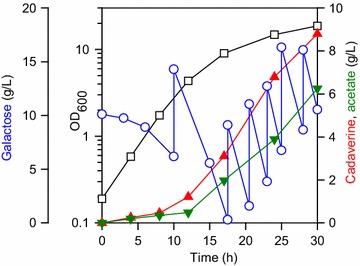
Time-course fed-batch fermentation profile for the DHK4 strain. The *left y-offset* and *right y-axis* represents galactose and cadaverine, acetate concentrations (g/L), respectively. The *left y-axis* represents OD_600_. The *x-axis* represents the culture time (h). Symbols: *open rectangle*, OD_600_; *open circle*, galactose; *closed upward triangle*, cadaverine; *closed downward triangle*, acetate

Fed-batch cultivation of the DHK4 strain was carried out to evaluate its performance in large-scale fermentation. We used a continuously stirred 5 L reactor with 2 L of initial medium volume with pH–stat mode. After inoculation, the cells immediately started to produce cadaverine. In the early phase during the first 12 h, the titer reached to 1.22 g/L and productivity was 0.101 g/L/h (Fig. 4). Cadaverine production accelerated with increased biomass, and productivity was observed during 12–30 h (0.421 g/L/h). Throughout the 30-h fermentation period, the final titer, yield, and productivity were significantly higher than flask-scale batch culture (8.80 g/L, 0.170 g/g, and 0.293 g/L/h, respectively).

## Discussion

By nature, microorganisms have evolved for rapid growth by fast utilization of the preferred carbon source. In addition, tight regulations on metabolic pathways by chemical-responsive transcription factors [29–31], riboswitches [32], and feedback inhibition [33] allow efficient carbon allocation and a reduction in wasted resources. This robustness of the metabolic network often incurs difficulties in the redesign of microorganisms as chemical-producing cell factories. Therefore, native metabolic networks should be altered through modification of fluxes toward both desired and unwanted pathways [20, 24, 34].

With the recent advances in synthetic biology, various predictive and quantitative genetic elements to control the expression of specific gene(s) are now available, and these tools can be efficiently utilized to redesign microorganisms. Depending on the required strength, the transcription machinery can be chosen from a synthetic promoter library [35–37]. Similarly, the 5′-UTR sequence, which is critical for translation initiation, can be precisely designed with consideration of the upstream coding sequence [26, 38]. As demonstrated in this study, we are now able to easily design and build synthetic pathways with maximum metabolic activity in a rational manner (galactose utilization and cadaverine production pathways). Moreover, the initial design can be further refined by modifying the design criteria as shown in the case of *cadA*. Accordingly, we successfully re-designed the metabolic pathways to produce cadaverine from galactose, resulting in the development of the DHK4 strain, which demonstrated high titer (8.80 g/L), yield (0.170 g/g), and productivity (0.293 g/L/h). These results are similar to those of glucose-based fermentation, demonstrating the highest yield ever studied in *E. coli*.

Although a fair amount of acetate (4.99 g/L) was still observed in the flask culture, it was significantly lower than that of the previous study [1]. When the fed-batch culture was conducted, 3.29-fold of cadaverine was produced compared to the batch culture while only 1.26-fold of acetate was accumulated. It is plausible that controlled aeration to maintain the saturated dissolved oxygen level in a bioreactor might help to enhance the activity of the electron transport chain for synthesizing ATP [39]. In addition, using synthetic and controllable genetic parts, carbon flux might be increasingly accelerated toward cadaverine formation even with reduced acetate formation. These results support the engineered DHK4 strain, which led to improved cellular performance with an increased yield. Alternatively, chromosomal deletion of *ackA*-*pta*, a main pathway for acetate production, is a possible trial as previously described [40, 41]. However, this deletion should be carefully applied as it sometimes causes reduced target chemical production with changes in growth pattern, although acetate production is significantly decreased [16]. Alternatively, replenishment of a key intermediate in the TCA cycle such as oxaloacetate can be an option to directly supply a precursor for cadaverine production and energy generation [42]. Therefore, optimal carbon flux distribution around the phosphoenolpyruvate–oxaloacetate node can presumably enhance the production of cadaverine from galactose. Optimization might be achieved by controlling the activity of anaplerotic enzymes such as PEP carboxylase [38].

## Conclusions

In summary, we re-constructed the metabolic pathway of *E. coli*, using synthetic expression designs to efficiently produce cadaverine from galactose. Additional improvement on cadaverine production was achieved by removing cadaverine degradation and re-uptake pathways. From fed-batch fermentation, our engineered strain showed 8.80 g/L of cadaverine production with 0.170 g/g of yield and 0.293 g/L/h of productivity. To our best knowledge, this is the first report to produce cadaverine from galactose with the yield for cadaverine production being the highest compared to those of previous studies in engineered *E. coli*.

## Methods

### Chemical reagents and oligonucleotides

Phusion DNA polymerase and restriction endonucleases were purchased from New England Biolabs (Ipswich, MA, USA). The oligonucleotides were synthesized by Cosmogenetech (Seoul, Korea) and listed in Additional file 1: Table S2. The amplified PCR products were purified using the GeneAll^R^ Expin™ Gel SV kit (GeneAll Biotechnology, Seoul, Korea). Genomic DNA was extracted using the GeneAll^R^ Exgene™ Cell SV kit. Plasmids were prepared using the AccuPrep^R^ Nano-Plus Plasmid Mini Extraction Kit (Bioneer, Daejeon, Korea). Other chemical reagents used in this study were obtained from Sigma-Aldrich (St. Louis, MO, USA).

### Construction of strains and plasmids

The strains and plasmids used in this study are listed in Table 1. To construct the pACYC-Lys plasmid, the artificial lysine operon was designed using a strong constitutive promoter (BBa_J23100) and the synthetic 5′-UTR predicted by the UTR Designer (http://sbi.postech.ac.kr/utr_designer) to maximally express genes related to lysine pathways [26, 38]. The *lysC*, *asd*, *dapA*, *dapB*, and *lysA* genes were amplified from the genomic DNA of *E. coli* W3110 by PCR reaction with V-lysC-F/V-lysC-R, V-asd-F/V-asd-R, V-dapA-F/V-dapA-R, V-dapB-F/V-dapB-R, and V-lysA-F/V-lysA-R primer pairs. The *ddh* was also amplified from the genomic DNA of *C. glutamicum* with V-ddh-F and V-ddh-R primers. Site-directed mutagenesis on *dapA* and *lysC* was conducted with P-dapA-F/P-dapA-R and P-lysC-F/P-lysC-R primer pairs via TA cloning. The amplified *asd* fragment was digested with *Xba*I and *Sph*I endonuclease and this was inserted into the pACYCDuet plasmid. Likewise, *dapA* ^*fbr*^, *dapB*, *ddh,* and *lysA* fragments were digested with *Sph*I and *Sac*I, *Sac*I and *Not*I, *Not*I and *Kpn*I, and *Kpn*I and *Pac*I endonucleases, respectively, and sequentially inserted into proper cloning sites to yield the pACYC-Lys plasmid. To construct the pET-cadA plasmid, *cadA* was amplified from the genomic DNA of *E. coli* W3110 using the V-cadA-F/V-cadA-R pair. It was then digested with *Bam*HI and *Pac*I endonucleases and inserted into a pETduet plasmid.

**Table 1:** Strains and plasmids used in this study

Name	Relevant characteristics	Source
Strains		
Mach1-T1R	F^−^ φ80(*lac*Z)ΔM15 Δ*lac*X74 *hsd*R(r_K_-mK +) Δ*rec*A1398 *end*A1 *ton*A	Invitrogen
W3110	F- λ- rph-1 IN(rrnD, rrnE)1	ATCC 27325
DHK1	W3100 rpsL (A128G)/pET-cadA	This study
DHK2	DHK1 P_galR_-UTR_galR_-*galR*-P_lysA_-UTR_lysA_-*lysA*:: P_BBa___J23100_-SynUTR_asd_-*asd*-P_BBa___J23100_-SynUTR_dapA_-*dapA* ^*fbr*^-P_BBa___J23100_-SynUTR_dapB_-*dapB*-P_BBa___J23100_-SynUTR_ddh_-*ddh*-P_BBa___J23100_-SynUTR_lysA_-*lysA* P_lysC_-UTR_lysC_-*lysC*:: P_BBa___J23100_-SynUTR_lysC_-*lysC* ^*fbr*^	This study
DHK3	DHK2 *galETKM*:: P_BBa___J23100_-SynUTR_galE_-*galE*-_PBBa___J23100_-SynUTR_galT_-*galT*-_PBBa___J23100_-SynUTR_galK_-*galK*-_PBBa___J23100_-SynUTR_galM-_ *galM* P_galP_-UTR_galP_::P_BBa_J23100_-SynUTR_galP_ P_pgm_-UTR_pgm_::P_BBa_J23100_-SynUTR_pgm_	This study
DHK4	DHK3 Δ*speE* Δ*speG* Δ*ygjG* Δ*puuPA*	This study
Plasmids		
pKD46	Red recombinase expression vector, Amp^R^	[43]
pCP20	FLP expression vector, Amp^R^, Cm^R^	[43]
pETduet	Expression vector, ColE1 ori, Amp^R^	Novagen
pACYCduet	Expression vector, p15A ori, Cm^R^	Novagen
pACYC-Lys	p15A ori, Cm^R^, P_BBa___J23100_-SynUTR_asd_-*asd*- P_BBa___J23100_-SynUTR_dapA_-*dapA*- P_BBa___J23100_-SynUTR_dapB_-*dapB* ^*fbr*^- P_BBa___J23100_-SynUTR_ddh_-*ddh*- P_BBa___J23100_-SynUTR_lysA_-*lysA*	This study
pET-cadA	ColE1 ori, Amp^R^, P_BBa___J23100_-SynUTR(v3)_cadA_–*cadA*	This study
pACYC-galO	p15A ori, Cm^R^, FRT-Kan^R^-FRT-P_BBa_J23100_-SynUTR_galE_-*galE*-P_BBa_J23100_-SynUTR_galT_-*galT* **-**P_BBa_J23100_-SynUTR_galK_-*galK* **-**P_BBa_J23100_-SynUTR_galM_-*galM*	[15]

All chromosomal manipulations were conducted using the Lambda Red recombination system with either the *rpsL*-*neo* or FRT-*Kan* ^*R*^-FRT fragment with pKD46 and pCP20 plasmids as previously described [40, 43–45]. For recombination with an *rpsL*-*neo* fragment, an *rpsL* A128G mutation was inserted into strain W3110 by direct recombination with the P-A128G oligonucleotide. The DHK1 strain was constructed by transforming the pET-cadA plasmid. To develop the DHK2 strain, we deleted a chromosomal region from *galR* to *lysA* by integrating the *rpsL*-*neo* fragment amplified with D1-galR-F/D1-galR-R primers. The PCR fragment containing synthetic expression cassettes for *asd*, *dapA* ^*fbr*^, *dapB*, *ddh,* and *lysA* was prepared by amplification with O-lysO-F/O-lys-R primers, using pACYC-Lys as a template, and this was integrated into the aforementioned region. In the case of *lysC*, native *lysC* was deleted by inserting the *rpsL*-*neo* fragment amplified with D1-lysC-F/D1-lys-R primers. The amplified *lysC* ^*fbr*^ fragment amplified with O-lysC-F/O-lysC-R primers was then integrated into the original site.

To construct DHK3 strain, the native *galETKM* operon was deleted by inserting the FRT-*Kan* ^*R*^-FRT fragment amplified by D1-galETKM-F/D1-galETKM-R primers. Next, the refactored *galETKM* [15] was integrated by inserting the PCR fragment amplified with O-galETKM-F/O-galETKM-R primers, and pACYC-galO as a template. In addition, both *galP* and *pgm* were overexpressed using the FRT-*Kan* ^*R*^-FRT fragment amplified with O-galP-F/O-galP-R1/O-galP-R2 and O-pgm-F/O-pgm-R, respectively.

The DHK4 strain was developed by deleting competing pathways for cadaverine synthesis [1]. To delete the *speE*, *speG*, *ygjG*, and *puuPA* genes, proper *rpsL*-*neo* fragments were utilized following amplification with D1-speE-F/D1-speE-R, D1-speG-F/D1-speG-R, D1-ygjG-F/D1-ygjG-R, and D1-puuPA-F/D1-puuPA-R pairs. After confirmation of deletion, *rpsL*-*neo* was removed by another direct recombination using D2-speE, D2-speG, D2-ygjG, and D2-puuPA oligonucleotides.

### Medium and culture conditions

For cadaverine production, the cells were aerobically cultivated in modified R/2 medium supplemented with 20 g/L galactose, 3 g/L (NH_4_)_2_SO_4_, 2 g/L (NH_4_)_2_HPO_4_, 6.75 g/L KH_2_PO_4_, 0.85 g/L citric acid, 0.7 g/L MgSO_4_·7H_2_O, and 5 mL/L trace metal solution [1]. The trace metal solution contained 10 g/L FeSO_4_·7H_2_O, 2.0 g/L CaCl_2_·2H_2_O, 2.2 g/L ZnSO_4_·7H_2_O, 0.5 g/L MnSO_4_, 1.0 g/L CuSO_4_·5H_2_O, 0.1 g/L (NH_4_)_6_Mo_7_O_2_·4H_2_O, and 0.02 g/L Na_2_B_4_O_7_·10H_2_O [46]. The pH was adjusted to 6.8 using 10 M KOH. For selection pressure, 100 μg/mL ampicillin was added to the culture medium as necessary.

The flask-scale batch culture was prepared by inoculating a single colony from an LB (Lysogeny broth) plate to 3 mL of modified R/2 media. After overnight culture, the initial seed cultures were re-inoculated to 3 mL of the same medium with optical density (OD_600_) 0.05 for refreshing. When the OD_600_ reached 0.8–1.0, cells were transferred into 25 mL of fresh medium with OD_600_ of 0.05. The cells were cultured at 37 °C with continuous shaking (250 rpm). The pH of the medium was adjusted to 6.8 every 6 h with 10 M KOH solution. The culture samples were periodically withdrawn and frozen at −80 °C until analysis. All experiments for cell culture were conducted in biological duplicate. The cell mass was measured using a UV-1700 spectrophotometer (Shimadzu, Kyoto, Japan) at a wavelength of 600 nm (OD_600_).

For the fed-batch culture, seeds were prepared by the same method as described above. After refreshing, the cells were transferred to 2 L of modified R/2 medium containing 10 g/L galactose in a 5-L jar fermenter with an initial OD_600_ of 0.2. The culture broth was continuously stirred at 500 rpm, and sterile air was provided at a flow rate of 2 vvm. The pH of the culture medium was automatically maintained at 6.8 with 10 M KOH solution. The temperature of the medium was also automatically controlled at 37 °C. The feeding solution [1] containing 300 g/L galactose, 8 g/L MgSO_4_·7H_2_O, and 115 g/L (NH_4_)_2_SO_4_ was used to supplement the carbon and nitrogen sources. A small amount of antifoam 204 (less than 0.1% v/v) was intermittently added to prevent foaming during fermentation.

### Analytical methods

To quantify the metabolites, the UltiMate™ 3000 analytical HPLC system (Dionex, Sunnyvale, CA, USA) was utilized. The concentration of galactose and acetate was analyzed using the Aminex HPX-87H column (Bio-Rad Laboratories, Richmond, CA, USA) with 5 mM H_2_SO_4_ as a mobile phase at a flow rate of 0.6 mL/min at 14 °C. The signals were monitored using a Shodex RI-101 detector (Shodex, Klokkerfaldet, Denmark).

For cadaverine analysis, the concentration was determined using a pre-column *o*-phthalaldehyde derivatization method coupled with reverse-phase liquid column chromatography (Acclaim 120 C18; Dionex, Sunnyvale, CA, USA) [47]. The derivatized cadaverine was eluted at a flow rate of 0.8 mL/min with a combination of eluent A (0.1 M sodium acetate in 55% methanol, pH 7.2) and eluent B (100% methanol) [48]. Samples with high cadaverine concentration were diluted to a concentration of less than 1 g/L for an accurate analysis. The signal was monitored using a UV–Vis diode array detector at a wavelength of 338 nm.

## Additional file

**Additional file 1.** Supplementary tables.

## Abbreviations

UTR: untranslated regions
ATP: adenosine triphosphate
PCR: polymerase chain reaction
*Kan*^*R*^: kanamycin resistance gene
OD: optical density
