# Supplementary material for: Synthetic redesign of Escherichia coli for cadaverine production from galactose
Source: Biotechnol Biofuels. 2017 Jan 21;10:20. doi: 10.1186/s13068-017-0707-2 (PMC5251296; doi:10.1186/s13068-017-0707-2)
Supplement: Supplementary file 1 — Additional file 1. Supplementary tables. [file 13068_2017_707_MOESM1_ESM.doc]

**Supplementary Table**

**Supplementary Table S1.** Synthetic 5’-UTRs designed for the expression of genes in galactose utilization and cadaverine production

| **Gene** | **Synthetic 5’-UTR** | **N-terminal 35 bp** | **dG_SD_**  (kcal/mol) | **dG_start_**  (kcal/mol) | **dG_spacing_**  (kcal/mol) | **dG_direct_**  (kcal/mol) | **dG_indirect_**  (kcal/mol) | **dG_UTR_**  (kcal/mol) | **Predicted Expression Level** (a. u.) |
| --- | --- | --- | --- | --- | --- | --- | --- | --- | --- |
| *galE* | attatcgacataaggaggttcgc | atgagagttctggttaccggtggtagcggttacat | -14.98 | -1.19 | 0.01 | -3.10 | -7.70 | -10.77 | 3,289,607.02 |
| *galT* | actcgcgttaaggaggcttatc | atgacgcaatttaatcccgttgatcatccacatcg | -14.18 | -1.19 | 0.00 | -8.00 | -3.00 | -9.88 | 1,994,467.19 |
| *galK* | acacctcaaggaggaggctt | atgagtctgaaagaaaaaacacaatctctgtttgc | -14.58 | -1.19 | 0.00 | -5.90 | -4.60 | -10.53 | 2,868,880.67 |
| *galM* | agataactagactcttataaaggaggaccca | atgctgaacgaaactcccgcactggcacccgatgg | -14.58 | -1.19 | 0.01 | -4.00 | -7.30 | -10.12 | 2,286,959.28 |
| *galP* | atttcatattaataaggaggaataa | atgcctgacgctaaaaaacaggggcggtcaaacaa | -14.88 | -1.19 | 0.01 | -1.70 | -5.90 | -12.27 | 7,611,940.33 |
| *pgm* | aggagtcgtaaaggaggagatacta | atggcaattcataatagagcaggtcaaccagcaca | -14.58 | -1.19 | 0.67 | -4.50 | -4.10 | -10.80 | 3,351,497.77 |
| *asd* | aaaataagtcaaaggagcatctata | atgaaaaatgttggttttatcggctggcgcggtat | -10.38 | -1.19 | 0.29 | -1.40 | -5.20 | -7.99 | 693,793.12 |
| *dapA* | aattcagaaaaaaggagcatcatct | atgttcacgggaagtattgtcgcgattgttactcc | -10.38 | -1.19 | 0.29 | -4.80 | -2.80 | -7.49 | 524,541.12 |
| *dapB* | tacattggcggagggaagtaaggtc | atgcatgatgcaaacatccgcgttgccatcgcggg | -12.78 | -1.19 | 2.40 | -2.60 | -11.10 | -4.73 | 111,914.73 |
| *ddh* | acaccaaaacaaaggagcatcccac | atgaccaacatccgcgtagctatcgtgggctacgg | -10.38 | -1.19 | 0.29 | -1.50 | -10.20 | -5.44 | 166,660.50 |
| *lysA* | gatacacaacaaaggagcatccctc | atgccacattcactgttcagcaccgataccgatct | -10.38 | -1.19 | 0.29 | -3.70 | -2.10 | -8.39 | 867,741.47 |
| *lysC* | aattccatatactaaggaggttcga | atgtctgaaattgttgtctccaaatttggcggcac | -14.98 | -1.19 | 0.01 | -1.60 | -6.70 | -12.02 | 6,618,661.18 |
| *cadA* | (v1) agcatagagacctaaggagtataga | atgaacgttattgcaatattgaatcacatgggggt | -10.98 | -1.19 | 0.01 | -1.40 | -1.40 | -10.77 | 3,289,607.02 |
| *cadA* | (v2) aaggacacctcagaaggagcacctc |  | -10.48 | -1.19 | 0.01 | -4.70 | -9.10 | -4.77 | 114,746.28 |
| *cadA* | (v3) agtggctcttacaaaggagccccct |  | -10.38 | -1.19 | 0.01 | -9.60 | -12.20 | -0.67 | 11,583.64 |

^a^5’ UTRs for *galE*, *galT*, *galK*, *galM*, *galP*, and *pgm* were originally designed in our previous study (Lim et al., Bioresour Technol. 2013, 135:564-7).

**Supplementary Table S2.** Primers used in this study.

| **Name^a^** | **Sequence (5’-3’)^b,c^** |
| --- | --- |
| V-lysC-F | caccgaGGATCCttgacggctagctcagtcctaggtacagtgctagcaattccatatactaaggaggttcgaatgtctgaaattgttgtctccaaatttg |
| V-lysC-R | ttacagGGTACCttactcaaacaaattactatgcagtttttg |
| V-asd-F | caccgaTCTAGAttgacggctagctcagtcctaggtacagtgctagcaaaataagtcaaaggagcatct |
| V-asd-R | ttacagGCATGCttacgccagttgacgaagcat |
| V-dapA-F | caccgaGCATGCttgacggctagctcagtcctaggtacagtgctagcaattcagaaaaaaggagcatcatctatgttcacgggaagtattgtcg |
| V-dapA-R | ttacagGAGCTCttacagcaaaccggcgtgc |
| V-dapB-F | caccgaGAGCTCttgacggctagctcagtcctaggtacagtgctagctacattggcggagggaagtaaggtcatgcatgatgcaaacatccg |
| V-dapB-R | ttacagGCGGCCGCttacaaattattgagatcaagtacatctcg |
| V-ddh-F | caccgaGCGGCCGCttgacggctagctcagtcctaggtacagtgctagcacaccaaaacaaaggagcatcccacatgaccaacatccgcgtagc |
| V-ddh-R | ttacagGGTACCttagacgtcgcgtgcgatc |
| V-lysA-F | caccgaGGTACCttgacggctagctcagtcctaggtacagtgctagcgatacacaacaaaggagcatccctcatgccacattcactgttcagc |
| V-lysA-R | ttacagTTAATTAAttaaagcaattccagcgccagtaatt |
| V-cadA-F | caccgaGGATCCttgacggctagctcagtcctaggtacagtgctagcagtggctcttacaaaggagccccctatgaacgttattgcaatattgaatc |
| V-cadA-R | ttacagTTAATTAAttattttttgctttcttctttcaatacc |
| O-lysO-F | ctctgggatcaccactttagcaacctgaagccaaacgccaccagcggtcttgacggctagctcagtccta |
| O-lysO-R | ctttttatgatgtggcgtaatcataaaaaagcacttatctggagtttgttttaaagcaattccagcgcca |
| O-lysC-F | Gtagcgccagtcacagaaaaatgtgatggttttagtgccgttagcgtaatgcatgaccggcgcgatgc |
| O-lysC-R1 | gctcctttgacttttagggctagcactgtacctaggactgagctagccgtcaagctcagcggatctcatgcgc |
| O-lysC-R2 | aaatcagctacgctggtaccgccaaatttggagacaacaatttcagacattatagatgctcctttgacttttagggctagc |
| O-galETKM-F | cttagcaccctctccggcc |
| O-galETKM-R | ctggtgatttgaacaatatgagataaagcc |
| O-galP-F | gcccgcacaataacatcattcttcctgatcacgtttcaccgcagattatcccgcatgaccgcgcgatgc |
| O-galP-R1 | cctccttattaatatgaaatgctagcactgtacctaggactgagctagccgtcaacgcgacgacaggcacatgcg |
| O-galP-R2 | gcagacgaaaaacgtcattgccttgtttgaccgcccctgttttttagcgtcaggcatttattcctccttattaatatgaaatgctagcac |
| O-pgm-F | cgatgcaattcccggcggaattgattgagaaggtttgcggaactatctaaacacatttaataaaaaaagggcggtcgcaagatcgcccttttttgcatgaccggcgcgatgc |
| O-pgm-R | taatattgcgccgtcagttgggcgacgttaatcaaatcactctgttgtgctggttgacctgctctattatgaattgccattagtatctcctcctttacgactcctgctagcactgtacctaggactgagctagccgtcaagctcagcggatctcatgcgc |
| P-dapA-F | aaggtttgtatcagtatttcaaagccatcg |
| P-dapA-R | cgatggctttgaaatactgatacaaacctt |
| P-lysC-F | gaagtgagcgtggcattaatccttgatacc |
| P-lysC-R | ggtatcaaggattaatgccacgctcacttc |
| P-A128G | atgcccgcaaaaacgtggcgtatgtactcgtgtatatactaccactcctagaaaaccgaactccgcgctgcgtaaagtatgccgtgttcgtctgactaacg |
| D1-galR-F | ctctgggatcaccactttagcaacctgaagccaaacgccaccagcggtcggcctggtgatgatggcgggatcg |
| D1-galR-R | ctttttatgatgtggcgtaatcataaaaaagcacttatctggagtttgtttcagaagaactcgtcaagaaggcg |
| D1-lysC-F | gactttggaagattgtagcgccagtcacagaaaaatgtgatggttttagtggcatgaccggcgcgatgc |
| D1-lysC-R | gacaagaaaatcaatacggcccgaaatatagcttccaggccatacagtatgctcagcggatctcatgcgc |
| D1-galETKM-F | cttagcaccctctccggccaacggttcgacgcatgcaggcatgaaaccgcccgcatgaccgcgcgatgc |
| D1-galETKM-R | ctggtgatttgaacaatatgagataaagccctcatgacgagggcgtaacacgcgacgacaggcacatgcg |
| D1-speE-F | attatgttgcgccctttttttacgggtgttaacaaaggaggtatcaacccggcctggtgatgatggcgggatcg |
| D1-speE-R | agattattaaagccatgcagtttcagttttttcaatttcttatcttctcctcagaagaactcgtcaagaaggcg |
| D1-speG-F | cgttattaccccctaacctgttattgatttaaggaatgtaaggacacgttggcctggtgatgatggcgggatcg |
| D1-speG-R | gccgtcgaacgggtttacaccatcaaaaatacgatcgattattattaatgtcagaagaactcgtcaagaaggcg |
| D1-ygjG-F | cgatcgcagccggagtggcgcaatccctgcaatacttaaatcggtatcatggcctggtgatgatggcgggatcg |
| D1-ygjG-R | gtcgtataaaaagatcggatggcgacgtcgtatcgccatccgatttgatatcagaagaactcgtcaagaaggcg |
| D1-puuPA-F | atattttacgctttgataacgagcggaaaacaaaccaaaggcgaagaatcggcctggtgatgatggcgggatcg |
| D1-puuPA-R | tggcgcggcgcgttaccctcaggcaggataatgcgccgcgcatccgactatcagaagaactcgtcaagaaggcg |
| D2-speE | attatgttgcgccctttttttacgggtgttaacaaaggaggtatcaacccggagaagataagaaattgaaaaaactgaaactgcatggctttaataatct |
| D2-speG | cgttattaccccctaacctgttattgatttaaggaatgtaaggacacgttcattaataataatcgatcgtatttttgatggtgtaaacccgttcgacggc |
| D2-ygjG | cgatcgcagccggagtggcgcaatccctgcaatacttaaatcggtatcattatcaaatcggatggcgatacgacgtcgccatccgatctttttatacgac |
| D2-puuPA | atattttacgctttgataacgagcggaaaacaaaccaaaggcgaagaatctagtcggatgcgcggcgcattatcctgcctgagggtaacgcgccgcgcca |
| C-galR-F | gcacgacgactcttcgccag |
| C-galR-R | gggcgatgtctttacccagcagg |
| C-pgm-F | gctttcggatgaatacgcagagc |
| C-pgm-R | cggacagggcgtgagtatc |
| C-galP-F | ggtcgtgaacatttcccgtg |
| C-galP-R | gaacatcatggagcttacgaccc |
| C-galETKM-F | cgctgaatcgccagcttatccg |
| C-galETKM-R | ccagaaccagcttagttacagcc |
| C-speE-F | ggcccgcgattccttaagc |
| C-speE-R | tataaccgtcgcgctcttcg |
| C-speG-F | ccgcttttgataaccgcgataaatg |
| C-speG-R | caacgggttggtaatgtaaaacagag |
| C-ygjG-F | catacccgacaaaaaccgtgc |
| C-ygjG-R | ggaatgcatgctgttgtgtgc |
| C-puuPA-F | ccatgctcaatctcacaaagtgg |
| C-puuPA-R | cattatttgcccctggggttaatg |

^a^Names beginning with “C” indicate primers used to check homologous recombination.

^b^Capital letters indicate restriction sites.

^c^Underlined letters indicate homologous sequences for recombination.
